# Supplementary material for: Prenatal Exposure to Parabens Affects Birth Outcomes through Maternal Glutathione S-Transferase (GST) Polymorphisms: From the Mothers and Kids Environmental Health (MAKE) Study
Source: Int J Environ Res Public Health. 2021 Mar 15;18(6):3012. doi: 10.3390/ijerph18063012 (PMC7998485; doi:10.3390/ijerph18063012)
Supplement: Supplementary file 1 [file ijerph-18-03012-s001.pdf]

Table S1. General Characteristics of overall study and study population.

**Characteristics**	**N (%)**	
	**Total**	**Study population**
Total	236	177
Maternal Age (years)		
<35	171 (72.46)	125 (70.62)
≥35	65 (27.54)	52 (29.38)
Pre-pregnancy BMI (kg/m2)		
<25.0	202 (85.59)	152 (85.88)
≥25.0	34 (14.41)	25 (14.12)
Past history of alcohol consumption		
Yes	189 (80.08)	139 (78.53)
No	47 (19.92)	38 (21.47)
Past history of smoking		
Yes	208 (88.14)	159 (89.83)
No	28 (11.86)	18 (10.17)
Genotype		
GSTM1		
Present	129 (54.89)	81 (45.76)
Null	106 (45.11)	96 (54.24)
GSTT1		
Present	136 (57.87)	83 (46.89)
Null	99 (42.13)	94 (53.11)

Table S2. Regression model testing the main and interactive effects of paraben concentrations and GSTM1/GSTT1 genotype on birth outcomes [a].

Interaction	Birth Weight(g)		Gestational age(wks)		Birth height(cm)	
	**B (SE) [a]**	***p*-Value**	**B (SE) [a]**	***p*-Value**	**B (SE) [a]**	***p*-Value**
GSTM1						
Methyl paraben	252.686 (79.535)	0.002	0.472 (0.278)	0.091	0.429 (0.487)	0.380
GSTM1 × methylparaben p-interaction		0.002		0.146		0.065
Ethyl paraben	16.589 (80.016)	0.836	0.180 (0.272)	0.509	−0.121 (0.48)	0.559
GSTM1 × ethylparaben p-interaction		0.825		0.623		0.807
propylparaben	198.317 (80.109)	0.014	0.580 (0.275)	0.036	0.630 (0.480)	0.816
GSTM1 × propylparaben p-interaction		0.014		0.041		0.032
GSTT1						
Methyl paraben	49.655 (78.554)	0.528	0.194 (0.268)	0.469	-0.085 (0.476)	0.859
GSTT1 × methylparaben p-interaction		0.602		0.937		0.770
Ethyl paraben	30.348 (82.709)	0.714	0.162 (0.281)	0.566	0.368 (0.501)	0.464
GSTT1 × ethylparaben p-interaction		0.679		0.672		0.122
propylparaben	24.611 (81.418)	0.763	0.286 (0.277)	0.303	-0.004 (0.48)	0.993
GSTT1 × propylparaben p-interaction		0.523		0.543		0.849

[a] Adjusted for mother's age, pre-pregnancy BMI, past history of alcohol consumption, past history of smoking and child's gestational age at delivery, gender, and parity. [b] Paraben concentrations were divided into 2 groups (≥75th percentile and <75th percentile).
